# Supplementary material for: Pharmacists’ Knowledge of Factors Associated with Dementia: The A-to-Z Dementia Knowledge List
Source: Int J Environ Res Public Health. 2021 Sep 22;18(19):9934. doi: 10.3390/ijerph18199934 (PMC8508463; doi:10.3390/ijerph18199934)
Supplement: Supplementary file 1 [file ijerph-18-09934-s001.zip › ijerph-1323362-sup.pdf]

Questionnaire Questions	Choose one of the 4 options*			
1. Hearing loss is:	**Risk Factor**	Protective Factor	Non-Associated Factor	I don't know
2. Chronic benzodiazepine use is:	**Risk Factor**	Protective Factor	Non-Associated Factor	I don't know
3. Elevated levels of total cholesterol constitute a:	**Risk Factor**	Protective Factor	Non-Associated Factor	I don't know
4. Listening to music daily is:	Risk Factor	Protective Factor	**Non-Associated Factor**	I don't know
5. Cognitive stimulation constitutes a:	Risk Factor	**Protective Factor**	Non-Associated Factor	I don't know
6. Reading daily constitutes a:	Risk Factor	**Protective Factor**	Non-Associated Factor	I don't know
7. Playing a musical instrument is usually:	Risk Factor	**Protective Factor**	Non-Associated Factor	I don't know
8. Watching television daily is:	Risk Factor	Protective Factor	**Non-Associated Factor**	I don't know
9. Suffering from depression years before (more than 10) the development of dementia is a:	**Risk Factor**	Protective Factor	Non-Associated Factor	I don't know
10. High levels of physical activity constitute a:	Risk Factor	**Protective Factor**	Non-Associated Factor	I don't know
11. The use of anticholinergic drugs constitutes a:	**Risk Factor**	Protective Factor	Non-Associated Factor	I don't know
12. Having a family history of some type of dementia is:	**Risk Factor**	Protective Factor	Non-Associated Factor	I don't know
13. Maintaining body hygiene and cleanliness is:	Risk Factor	Protective Factor	**Non-Associated Factor**	I don't know
14. Controlling systolic blood pressure in middle-aged people is:	Risk Factor	**Protective Factor**	Non-Associated Factor	I don't know
15. Brain trauma constitutes a:	**Risk Factor**	Protective Factor	Non-Associated Factor	I don't know
16. A high adherence to the Mediterranean diet is a:	Risk Factor	**Protective Factor**	Non-Associated Factor	I don't know
17. Obesity, understood as a body mass index greater than 30 kg/m^2, is a:	**Risk Factor**	Protective Factor	Non-Associated Factor	I don't know
18. Jobs with a higher cognitive level constitute a:	Risk Factor	**Protective Factor**	Non-Associated Factor	I don't know
19. To be an only child is:	Risk Factor	Protective Factor	**Non-Associated Factor**	I don't know
20. Social isolation is a:	**Risk Factor**	Protective Factor	Non-Associated Factor	I don't know
21. Memory complaints constitute a:	**Risk Factor**	Protective Factor	Non-Associated Factor	I don't know
22. A high level of education constitutes a:	Risk Factor	**Protective Factor**	Non-Associated Factor	I don't know
23. Metabolic syndrome is a:	**Risk Factor**	Protective Factor	Non-Associated Factor	I don't know
24. Tobacco use constitutes a:	**Risk Factor**	Protective Factor	Non-Associated Factor	I don't know

25. Occupational therapy in the elderly population is a:	Risk Factor	**Protective Factor**	Non-Associated Factor	I don’t know
26. Being prone to cold sores is:	**Risk Factor**	Protective Factor	Non-Associated Factor	I don’t know
27. Using the internet or social networks from any technological device is:	Risk Factor	**Protective Factor**	Non-Associated Factor	I don’t know
28. Manual work is:	Risk Factor	Protective Factor	**Non-Associated Factor**	I don’t know
29. Being a woman constitutes a:	**Risk Factor**	Protective Factor	Non-Associated Factor	I don’t know
30. That people acquire a proactive attitude of prevention regarding the modifiable factors associated with dementia is:	Risk Factor	**Protective Factor**	Non-Associated Factor	I don’t know
31. Sleeping less than 6 h a day:	**Risk Factor**	Protective Factor	Non-Associated Factor	I don’t know
32. Sleeping more than 9 h a day:	**Risk Factor**	Protective Factor	Non-Associated Factor	I don’t know
33. Refraining from drinking alcohol is:	Risk Factor	**Protective Factor**	Non-Associated Factor	I don’t know
34. Living in the countryside or in rural areas is:	Risk Factor	**Protective Factor**	Non-Associated Factor	I don’t know
35. Taking anti-inflammatory drugs and/or having inflammatory diseases under control is:	Risk Factor	**Protective Factor**	Non-Associated Factor	I don’t know
36. Speaking several languages is:	Risk Factor	**Protective Factor**	Non-Associated Factor	I don’t know

* Correct answer in bold.
